# Substrate-Influenced Thermo-Mechanical Fatigue of Copper Metallizations: Limits of Stoney’s Equation

**DOI:** 10.3390/ma10111287

**Published:** 2017-11-09

**Authors:** Stephan Bigl, Stefan Wurster, Megan J. Cordill, Daniel Kiener

**Affiliations:** 1 Department of Materials Physics, Montanuniversität Leoben, Jahnstrasse 12, 8700 Leoben, Austria; stephan_bigl@hotmail.com (S.B.); stefan.wurster@unileoben.ac.at (S.W.); 2 Erich Schmid Institute of Materials Science, Austrian Academy of Sciences, Jahnstrasse 12, 8700 Leoben, Austria; megan.cordill@oeaw.ac.at

**Keywords:** thin films, stress, thermo-mechanical

## Abstract

Rapid progress in the reduction of substrate thickness for silicon-based microelectronics leads to a significant reduction of the device bending stiffness and the need to address its implication for the thermo-mechanical fatigue behavior of metallization layers. Results on 5 µm thick Cu films reveal a strong substrate thickness-dependent microstructural evolution. Substrates with *h_s_* = 323 and 220 µm showed that the Cu microstructure exhibits accelerated grain growth and surface roughening. Moreover, curvature-strain data indicates that Stoney’s simplified curvature-stress relation is not valid for thin substrates with regard to the expected strains, but can be addressed using more sophisticated plate bending theories.

## 1. Introduction

Thermo-mechanical fatigue of metallic thin films has been one of the key concerns in microelectronics during a product’s lifecycle. The material damage and subsequent failure regarding functionality is often associated with film cracking, severe surface roughening, and void formation in the film interior. The driving force behind such microstructural changes is always connected to the evolving stresses in the film material. Stresses arise as a consequence of the substrate constraint and the different coefficients of thermal expansion (CTE) between the film and substrate. The cornerstone of experimental evaluation of film stresses is performed with the well-known Stoney Equation [1]. Although many authors have developed refined calculations for substrate bending [2,3,4,5,6], Stoney’s equation has been used unchanged for more than a century. The arising biaxial film stress, *σ_f_*, is derived by the simple expression:

(1) $$\sigma_{f}=\frac{E_{s}\;h_{s}^{2}\;\kappa }{6\left(1-\upsilon_{s}\right)h_{f}}$$

where *h_s_*, *E_s_*, and *υ_s_* are the thickness, elastic modulus, and Poisson’s ratio of the substrate, *h_f_* is the film thickness, and *κ* denotes the film/substrate curvature (inverse of radius). Since biaxial film stresses develop as a consequence of the thermal mismatch strain, *ε_m_*, then:

(2) $$\varepsilon_{m}≅\left(\alpha_{s}-\alpha_{f}\right)\Delta T$$

which is an invariant bound by the different CTE of the substrate and film, *α_s_* and *α_f_*, determination of stresses is only connected to the variation of the film/substrate curvature. 

In other words, for the same *σ_f_* the corresponding curvature is proportional to 1/*h_s_*^2^ and *h_f_*, and scales with the elastic properties of the substrate. To guarantee validity, Equation (1) is based on the main assumption that *h_f_* is much less than *h_s_* [7]. Thereby, the criterion, h* = *h_f_*/*h_s_* is not strictly defined, but rather arbitrarily chosen (often set to h* = 0.01, see [6]). In modern microelectronics, increasing product performance requires thinner and thinner substrates to decrease the vertical resistance and device volume, while keeping the metallization thicknesses similar [8,9]. In literature, numerous studies report the effect of film thickness on microstructural changes [10,11,12], but experimental work dealing with the influence of substrate thickness is very limited. Few studies have shown that, as a consequence of the increase of h*, large non-linear deformations and bifurcation (*κ_x_* ≠ *κ_y_*) of the substrate curvature can occur due to deposited films [13,14]. This raises the question of in which range Stoney’s approximation is still valid with regard to cyclic thermal fatigue testing. Therefore, in the present study, the influence of substrate thickness is shown by thermally cycling identical 5 µm thick Cu films on silicon substrates with different thicknesses.

## 2. Materials and Methods

Copper films with *h_f_* = 5 µm were electrodeposited using a Cu electrolyte on 725 µm thick, (100)-oriented silicon wafers. Afterwards, samples were annealed for 30 min in an inert/reducing atmosphere at 400 °C. For detailed information regarding the used copper film and its microstructure, see references [15,16], where the film used here was denoted as Film A. To achieve different substrate thicknesses, sample pieces of 5 × 10 mm were cut, mounted into sample holders, and wet-ground with a semi-automated lab tool (Struers TegraPol, Struers ApS, Ballerup, Denmark). This process enabled specimens with *h_s_* down to ~200 µm. Below that thickness, sample integrity (e.g., substrate cracking) becomes a major concern. For thermal cycling and subsequent microstructural analysis, samples with *h_s_* of 725, 541, 323, and 220 µm resulting in h* of 0.007, 0.009, 0.015, and 0.023, respectively, were used. Those samples were thermo-mechanically cycled in an infrared furnace between 170 °C and 400 °C in a formic gas atmosphere. To study the effect of varying substrate thickness on the thermo-mechanical behavior of Cu films, a site-specific microstructural tracking technique was applied throughout the thermal cycling process [17]. Using electron back scatter diffraction (EBSD) (EDAX, Weiterstadt, Germany) in combination with atomic force microscopy (AFM) (Veeco, Aschheim, Germany), the microstructural and topographical evolution of a specifically marked film surface area was studied. Furthermore, scanning electron microscope (SEM) (Zeiss, Oberkochen, Germany) images and focused ion beam (FIB) film (Zeiss, Oberkochen, Germany) cross-sections were used to substantiate experimental evidence. To guarantee an oxide-free film surface, specimens were subjected to 100 vol % acetic acid at 35–40 °C for 3 min. This enables the selective removal of any copper oxide and does not affect the actual Cu microstructure [18]. Experimental determination of the substrate curvature evolution was performed with a custom-built wafer curvature set up with a multiple optical beam sensor, kSA MOS (k-Space Associates, Inc., Dexter, MI, USA), with a heating rate of 10 °C/min. Experimental details regarding temperature calibration, settings of the used instruments, and its software packages can be found in references [17,19,20].

## 3. Results

**Figure 1:**
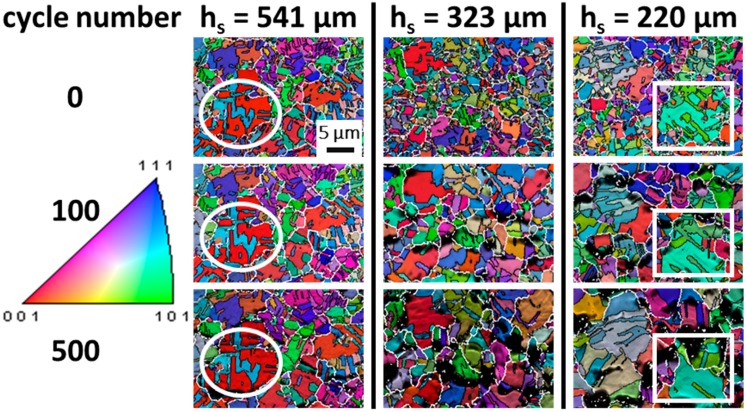
Microstructural evolution of 5-µm thick Cu films on silicon substrates with different thicknesses. The inverse pole figure-image quality (IPF-IQ) images display the changes with respect to the cycling stage of 0, 100, and 500 cycles. For thicker substrates, marginal microstructural changes in the grains (circular markings) were observed during thermal cycling, whereas the on thinner substrates the grains exhibited pronounced growth and twin migration (squares). Scale bar is valid for all electron back scatter diffraction (EBSD) images.

To quantitatively assess the effect of decreasing *h_s_* with respect to the cyclic thermo-mechanical behavior of the 5 µm thick Cu film, Table 1 presents the grain size (including twin boundaries) and standard deviation determined by EBSD for 0, 100, and 500 thermal cycles. For *h_s_* = 725 and 541 µm, an almost identical grain size evolution is present, where marginal grain growth during thermo-mechanical loading occurs. Below the 541 µm substrate thickness, significant grain growth could be observed. For *h_s_* = 323 µm, the grain size increased up to 4.3 ± 1.3 µm after 500 cycles, while for *h_s_* = 220 µm, the average grain size increased by a factor of two after 500 cycles. Figure 1 provides an overview of local microstructural changes for *h_s_* = 541, 323, and 220 µm. The inverse pole figure (IPF) images with overlaid image quality (IQ) in normal direction (ND) document the grain growth with respect to cycle number, where white lines indicate high angle grain boundaries (HAGB ≥ 15°) and thin black lines indicate primary twins (misorientation = 60°). For the Cu film on the thick substrate, *h_s_* = 541 µm, marginal microstructural changes could be observed during the cycling with respect to the initial stage. For instance, the ~ {100}//{212}-oriented parent/twin grain highlighted by a circle showed no essential plasticity during cycling, and in particular no twin boundary migration, as this controls grain growth in these films on standard 725 µm thick substrates [19]. On the contrary, the two thinner substrates (*h_s_* = 323 and 220 µm) revealed grain growth during the thermo-mechanical loading in combination with noticeable twin boundary migration as a plastic deformation mechanism. This was highlighted for *h_s_* = 220 µm in the squared section. The approximately {110}-oriented twinned grains showed distinct grain growth via HAGB migration as well as detwinning during cycling. 

**Figure 2:**
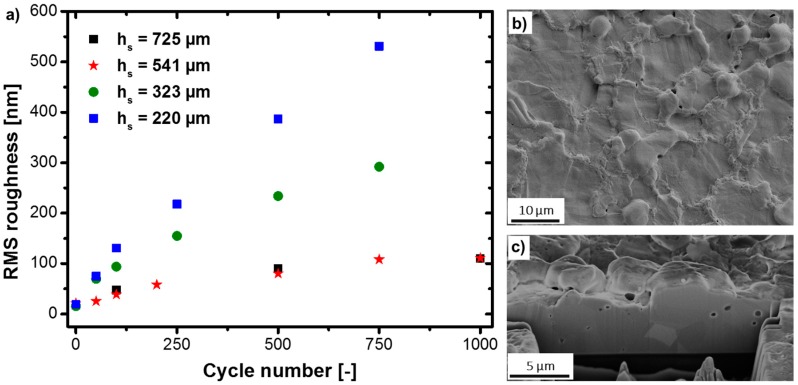
(**a**) Root mean squared (RMS) roughness evolution of a Cu film with *h_f_* = 5 µm on silicon substrates with different thicknesses (725, 541, 323, and 220 µm) as a function of cycle number; (**b**) SEM micrograph and (**c**) Focused ion beam (FIB) cross-section of a 5 µm thick film on *h_s_* = 220 µm after 750 thermal cycles.

A comparable trend was found when evaluating the roughness. Figure 2a illustrates the root mean squared (RMS) roughness evolution of the film surface with regard to a decreasing *h_s_*. An almost identical roughness evolution for *h_s_* = 725 and 541 µm was observed, in accordance with the grain size evolution in Table 1. Both films started with an initially very flat surface of about 20 nm RMS roughness, which increased up to 110 nm after 1000 cycles. For *h_s_* = 323 and 220 µm, a significant increase was observed. After 750 cycles, the RMS roughness of the Cu films on *h_s_* = 323 and 220 µm was increased by a factor of 3 and 5 compared to that on *h_s_* = 541 µm. The significant increase of surface roughening for thinner substrates was due to the formation of hillock-like features and voids on the surface (Figure 2b), as well as void formation at grain boundaries in the film interior (Figure 2c).

## 4. Discussion

The experimental findings show a significant substrate thickness dependency with regard to the thermo-mechanical fatigue behavior. The substrates with *h_s_* = 725 and 541 µm showed minimal grain growth (Table 1) and restricted surface roughening (Figure 2a), thus confirming results from a previous study on 725 µm thick substrates (denoted as Film A in that work) [19]. However, a distinctively different thermal fatigue behavior was observed for substrate thicknesses <541 µm. From a phenomenological point of view, it seems that at a certain h*, a threshold strain energy was overcome to enable the observed HAGB migration and twin boundary migration. Since the mismatch strain is only defined by the mismatch in CTE of the corresponding materials (see Equation 2), thermal film stresses were supposedly the same in every cycled sample and, hence, cannot cause the different thermal fatigue behavior. 

In a study by Chu et al. dealing with the reformulation of the Stoney formula, the author points out that the original problem of bending a composite beam (or plate) due to thermal stress will always result in a net bending moment as long as there is an elastic bending curvature [21]. In other words, the composite plate curvature results in a bending stress configuration which is opposite in sign to the generated thermal stress. The exact derivation not only points out the falsehood of Stoney’s simplified and arbitrarily chosen neutral axis position *b*, where *b* = 2*h_s_*/3 [1], but leads to a substantially different curvature-strain relation. For h* smaller than 0.1, Stoney’s formula would underestimate the corresponding bending curvature by a factor of ~2. (see Figure 6, [21]). Thereby, the curvature (inverse of the radius) is related to:

(3) $$\frac{1}{R}=\frac{12\frac{M_{f}}{M_{s}}\frac{h_{f}}{h_{s}}}{h_{s}\left(1+6\frac{M_{f}}{M_{s}}\frac{h_{f}}{h_{s}}\right)}\varepsilon_{m}$$

where *M_s_* and *M_f_* are the biaxial moduli of the substrate and film, respectively.

**Figure 3:**
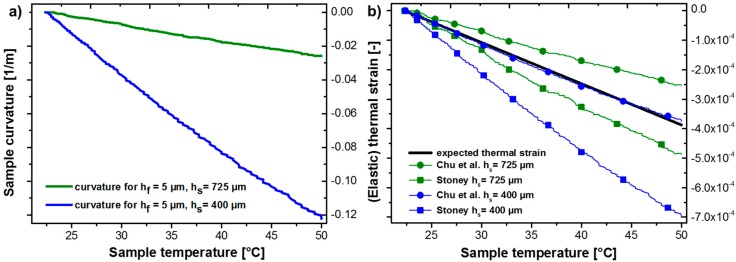
(**a**) Curvature evolution of a Cu film with *h_f_* = 5 µm on Si with *h_s_* = 725 and 400 µm; (**b**) Comparison of thermal strain evolution calculated following Stoney (squares) and Chu (circles) with regard to the measured curvature.

To validate the predictions of the actual bending curvature using Stoney and Chu curvature-(film) strain relations, the curvature evolution due to thermal mismatch was measured for substrate thicknesses of *h_s_* = 725 and 400 µm using substrates that were processed using semiconductor industry tools to exclude preparation artefacts. Both wafers had the same 5 µm thick Cu film on the top, and specimens of 10 × 10 mm in lateral dimension were used. In Figure 3a, the two corresponding sample curvatures are presented from room temperature up to 50 °C, where pure elastic strain development can be assumed. In both cases a linear curvature-temperature (strain) relationship was observed. The decrease of *h_s_* down to 400 µm increased the substrate curvature by a factor of ~5. For both configurations, the resulting thermal elastic strain in the copper film at any given temperature was nearly the same and followed Equation (2). We assume a constant CTE mismatch between Cu and Si of Δα = 14 ppm/K. Using the temperature data from the curvature measurements, the respective strain evolution is plotted as a thick black line in Figure 3b. It should be noted that due to the finite substrate thicknesses, a slight deviation to a smaller compressive film strain is present since a small portion of the mismatch strain is carried by the substrate. For the presented configurations this deviation can be neglected, because even for the thinner substrate the film thickness represents only about 1.25% of the total sample thickness (400 µm thick substrate, 5 µm thin film). As displayed in Table 2, a thermal strain of −3.9 × 10^−4^ and −65 MPa respectively (using *σ_f_* = *ε_m_ M_f_*, with *M_f_* = 168 GPa) at 50 °C is obtained. Rearranging Equations (1) and (3), we can calculate the strain from the curvature data using *M_s_* = 180.5 GPa. For a common wafer thickness, *h_s_* = 725 µm, final strains of −4.9 × 10^−4^ and −2.5 × 10^−4^ are obtained, which would result in −82 MPa and −42 MPa for Stoney and Chu, respectively. One could argue that this discrepancy regarding an overestimation for Stoney (and an underestimation for Chu’s relation) could simply be a sensitivity problem due to the small curvature evolution [22]. However, a clearer picture evolves for *h_s_* = 400 µm, where Stoney’s relationship would result in −7.0 × 10^−4^ and −118 MPa. This would overestimate the strain/stress by almost a factor of ~2, whereas the analysis of Chu et al. perfectly correlates with the developed stress by the predicted CTE mismatch of −65 MPa in the Cu film. 

These observations confirm that, in general, Stoney’s equation is only an approximation of the stress development for thin films on thick rigid substrates. For thin substrates, it leads to the wrong curvature-strain relationship, while the Chu et al. derivation, where the curvature is ~2·*κ*_stoney_, correlates well with our experimental observations. In fact, this emphasizes that the simplified model of having a net-zero bending moment with regard to the composite curvature is not correct. If we consider the stress fields proposed in reference [21], Equation (15) for the Cu layer, it becomes evident that a stress/strain gradient is directly proportional to the evolving curvature *κ*, scaling with the resulting bending curvature and the applied thermal strain *ε_m_*. This is similar to the bending strain relationship for flexible electronics, where the strain in the outer fiber (e.g., metallization layer) is proportional to its bending curvature [23]. With the decrease of *h_s_* and the understanding that the curvature is underestimated by a factor of ~2, significant strain gradients could locally lead to higher stresses in the surface region and cause an accelerated microstructure evolution, as evidenced in Figure 1 and Figure 2. Furthermore, the density of geometrically necessary dislocations *ρ_G_* is proportional to |*κ*|, which would give rise to a strain gradient in the bent lattice curvature [24]. 

**Figure 4:**
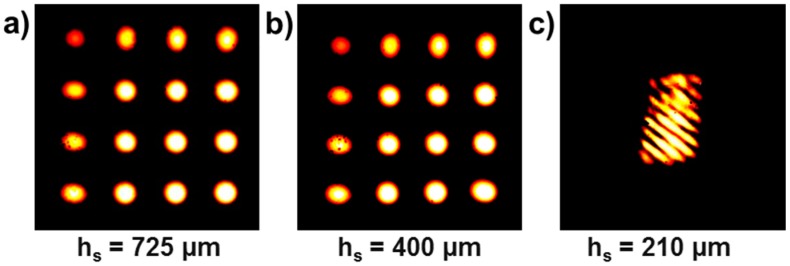
Detected multi-laser spot grid for a Cu film with *h_f_* = 5 µm at room temperature with a varying *h_s_* of (**a**) 725 µm; (**b**) 400 µm; and (**c**) 210 µm.

Furthermore, the CCD (charge couple device) detector images of the deflected laser beams in Figure 4 reveal that between *h_s_* = 400 µm and 210 µm, an abrupt “snap over” of the substrate curvature occurred, seen as a bifurcation of the sample curvature. Besides the impracticable measurement of the curvature evolution for *h_s_* = 210 µm, this observation points to the presence of large non-linear deformation. Focusing on the ellipsoidal shape of the detected laser spots suggests that the curvature is a function of position and that twist moments are present. Such effects rise inevitably in modern microelectronics, and are not considered in common analysis but can have a pronounced effect on the thermo-mechanical material behavior.

## 5. Conclusions

The detailed results regarding the microstructural evolution of 5 µm thick Cu films upon thermo-mechanical fatigue revealed a strong substrate thickness dependency. The findings showed that distinct grain growth coupled with twin migration and accelerated surface roughening was present for Cu films cycled on thin substrates with *h_s_* = 323 and 220 µm. The examination of Stoney’s curvature-strain relation using wafer curvature measurements showed distinct deviations between the expected and measured curvature. The significantly larger sample curvature in combination with expected plastic strain gradients in the film material can serve as an explanation for the accelerated material degradation. The observations with respect to non-uniform sample curvature and bifurcation for thinner substrates (e.g., 210 µm) further highlight the need for more detailed investigations of the actual limits of Stoney’s assumptions on small substrate deformation and its implications for microstructural effects on semiconductor thin film materials.

## Figures and Tables

**Table 1:** Grain size evolution of the Cu film with four different substrate thicknesses during cycling.

Cycle Number (-)	Grain Size (Including Twins) (µm)			
	*h_s_* = 725 µm	*h_s_* = 541 µm	*h_s_* = 323 µm	*h_s_* = 220 µm
0	2.4 ± 0.8	2.3 ± 0.7	2.5 ± 0.7	2.4 ± 0.8
100	2.7 ± 0.9	2.7 ± 0.8	3.6 ± 1.1	3.8 ± 1.2
500	2.8 ± 0.9	3.0 ± 1.0	4.3 ± 1.3	5.1 ± 1.6

**Table 2:** Comparison of the predicted strain and stress values in copper at 50 °C.

Method of Validation	*h_s_* = 725 µm		*h_s_* = 400 µm	
	Compressive *ε_m_*	Compressive *σ_f_*	Compressive *ε_m_*	Compressive *σ_f_*
**CTE mismatch**	−3.9 × 10^−4^	−65 MPa	−3.9 × 10^−4^	−65 MPa
**Stoney**	−4.9 × 10^−4^	−82 MPa	−7.0 × 10^−4^	−118 MPa
**Chu**	−2.5 × 10^−4^	−42 MPa	−3.7 × 10^−4^	−63 MPa

## References

[1] Stoney G.G. (1909). The Tension of metallic films deposited by electrolysis. Proc. R. Soc. Lond. Ser. A.

[2] Timoshenko S. (1925). Analysis of bi-metal thermostats. J. Opt. Soc. Am..

[3] Brenner A., Senderoff S. (1949). Calcuation of stress in electrodeposits from the curvature of a plated strip. J. Res. Natl. Bur. Stand.

[4] Timoshenko S.P., Woinowsky-Krieger S. (1959). Theory of Plates and Shells.

[5] Saul R.H. (1969). Effect of GaAs_x_P_1−x_ transistion zone on the perfection of GaP crystals grown by deposition onto GaAs substrates. J. Appl. Phys..

[6] Freund L.B., Floro J.A., Chason E. (1999). Extensions of the Stoney Formula for substrate curvature to configurations with thin substrates or large deformations. Appl. Phys. Lett..

[7] Janssen G., Abdalla M., van Keulen F., Pujada B., van Venrooy B. (2009). Celebrating the 100th anniversary of the Stoney equation for film stress: Developments from polycrystalline steel strips to single crystal silicon wafers. Thin Solid Films.

[8] Vobecky J., Rahimo M., Kopta A., Linder S. Exploring the Silicon Design Limits of Thin Wafer IGBT Technology: The Controlled Punch through (CPT) IGBT. Proceedings of the 2008 20th International Symposium on Power Semiconductor Devices and IC’s.

[9] Zoschke K., Wegner M., Wilke M., Jürgensen N., Lopper C., Kuna I., Glaw V., Röder J., Wünsch O., Wolf M.J. Evaluation of thin wafer processing using a temporary wafer handling system as key technology for 3D system integration. Proceedings of the 2010 Proceedings 60th Electronic Components and Technology Conference (ECTC).

[10] Zhang G., Schwaiger R., Volkert C., Kraft O. (2003). Effect of film thickness and grain size on fatigue-induced dislocation structures in Cu thin films. Philos. Mag. Lett..

[11] Zhang G., Volkert C., Schwaiger R., Wellner P., Arzt E., Kraft O. (2006). Length-scale-controlled fatigue mechanisms in thin copper films. Acta Mater..

[12] Kraft O., Schwaiger R., Wellner P. (2001). Fatigue in thin films: Lifetime and damage formation. Mater. Sci. Eng. A.

[13] Finot M., Blech I., Suresh S., Fujimoto H. (1997). Large deformation and geometric instability of substructures with thin-film deposits. J. Appl. Phys..

[14] Harper B.D., Chih-Ping W. (1990). A geometrically nonlinear model for predicting the intrinsic film stress by the bending-plate method. Int. J. Solids Struct..

[15] Wimmer A., Smolka M., Heinz W., Detzel T., Robl W., Motz C., Eyert V., Wimmer E., Jahnel F., Treichler R. (2014). Temperature dependent transistion of intragranular plastic to intergranular brittle failure in electrodeposited Cu micro-tensile samples. Mater. Sci. Eng. A.

[16] Bigl S., Schöberl T., Wurster S., Cordill M.J., Kiener D. (2016). Correlative microstructure and topography informed nanoindentation of copper films. Surf. Coat. Technol..

[17] Bigl S., Wurster S., Cordill M.J., Kiener D. (2015). Site specific microstructural evolution of themo-mechanically fatigued copper films. BHM Berg- und Hüttenmännische Monatshefte.

[18] Chavez K., Hess D. (2001). A novel method of etching copper oxide using acetic acid. J. Electrochem. Soc..

[19] Bigl S., Wurster S., Cordill M.J., Kiener D. (2016). Advanced characterisation of thermo-mechanical fatigue mechanisms of different copper film systems for wafer metallizations. Thin Solid Films.

[20] Bigl S., Heinz W., Kahn M., Schoenherr H., Cordill M.J. (2015). High-temperature characterization of silicon dioxide films with wafer curvature. JOM.

[21] Chu S. (1998). Elastic bending of semiconductor wafer revisited and comments on Stoney’s equation. J. Electrochem. Soc..

[22] Chason E. (2005). Resolution and Sensitivity of Stress Measurements with the k-Space Multi-Beam Optical Sensor (MOS) System.

[23] Suo Z., Ma E., Gleskova H., Wagner S. (1999). Mechanics of rollable and foldable film-on-foil electronics. Appl. Phys. Lett..

[24] Fleck N., Hutchinson J. (1997). Strain gradient plasticity. Adv. Appl. Mech..

